# Tuberculosis and Its Relation to the Veterinarian1Read before the Veterinary Medical Society of the Ontario Veterinary College.

**Published:** 1898-01

**Authors:** C. W. Fisher

**Affiliations:** Class ’98, Ontario Veterinary College


### TUBERCULOSIS AND ITS RELATION TO THE VETERINARIAN.[1]

By C. W. Fisher.

CLASS '98, ONTARIO VETERINARY COLLEGE.

Tuberculosis in general is too broad a subject for consideration in a paper of this kind. We will, however, endeavor to consider a few of the points of special interest to the veterinarian.

Being a topic of much general discussion at the present time, every veterinarian should become as familiar with the subject as possible. As so much has been written and published on tuberculosis in regard to its history, etiology, and pathology, we will pass hastily over these and consider more carefully a few of the important points of interest to the veterinarian.

Tuberculosis is an infectious disease caused by the bacillus tuberculosis, characterized by the formation of tubercles in the various organs of the body. It is common to man and all animals. It

[1] Read before the Veterinary Medical Society of the Ontario Veterinary College.

is not a new disease; in fact, Hippocrates, 400 B.C., described abscesses and ulcers of the lungs which characterize the disease of to-day.

This paper will consider bovine tuberculosis principally, as it is of the most importance to our profession, bringing in that of the other animals only in relation to this. Bovine tuberculosis is of long standing in European countries, but is comparatively new in America. It was brought here in cattle imported for stock-purposes, and has gradually spread from the stock-farms throughout the country. Within the past few years, through the cheapness of transportation and the desire to improve their herds, people have exchanged cattle much more than formerly, purchasing especially from these infected stock-farms. This has caused the disease to spread very fast, thus giving some people the idea that it is a new disease, while others think it has always existed in our herds. It does not originate spontaneously, however poor the management of a herd may be. A veterinarian should acquaint himself with the predisposing tendencies, also the laws of building, so as to enlighten a client who may seek his advice and services. There is much in breeding that will act as a preventive to this and other diseases.

Bovine tuberculosis, being so insidious in its character of development, and affecting any of so many various organs of the body, is a very hard disease to diagnose by physical examination alone. It is, however, important for the veterinarian to be familiar with a few of the general symptoms, some of which may be recognized in well-developed cases. First, there may be a dry, hoarse cough, especially in the morning or when the animal is exercised ; upon auscultation the different râles and crepitation may be heard, or the absence of normal sounds. In advanced cases crepitation may be felt by carefully placing the hands on either side of the thorax ; the animal will now have a generally unthrifty appearance. There may be enlargement of any of the lymphatic glands, such as the supra-mammary, inguinal, pre-scapular or retropharyngeal ; also the udder may be affected. The appetite may be abnormal until late in the development, when it will gradually diminish, and the animal will emaciate quite rapidly ; there may also be present a chronic diarrhœa. The cough and respiratory symptoms will now be exaggerated, and the animal will have a hectic fever of from 1° to 3° F.

Many of the preceding symptoms may be present, and yet the veterinarian is not certain of his diagnosis of tuberculosis, as other diseases may give similar symptoms. The most common disease which the veterinarian may mistake from these symptoms for tuberculosis is chronic bronchitis, but with this there is usually more discharge from the nostrils.

The tuberculin-test is of the greatest value in diagnosing the disease. It consists in the subcutaneous injection of a small quantity of tuberculin, the taking of the temperature before or at the time of the injection, also during the period of from ten to twenty hours after injection, and the noting of physical as well as temperature reactions.

Tuberculin was first used as a diagnostic agent in veterinary practice in 1891 by Prof. W. Gutmann, of the Veterinary Institute, Dorpat, Russia; in America by Prof. W. L. Zuill, of the University of Pennsylvania, Veterinary Department. It is a glycerin extract of tubercle bacilli. Tuberculin is prepared by growing pure cultures of the bacillus in serum or other suitable media until highly concentrated. Glycerin and carbolic acid are then added, and the whole passed through a porcelain filter to remove the bacilli, after which the liquid is heated for a time to kill the germs if any are present. The liquid is then concentrated by evaporation in a vacuum until of the proper strength.

The temperature reaction is explained by Gamaleia in this way: the toxins of the tubercle bacilli in the tuberculin have a poisonous action on the elements of the tubercle, causing necrobiosis. The ptomaïns resulting from the decomposition of the elements excite a local exudative inflammation and leucocytic infiltration. This local action results in the breaking up and elimination of the tubercular foci. The febrile reaction must be attributed to the absorption of the necrosed tissues.

The Koch tuberculin is usually diluted 1 : 10 in a 1 per cent. solution of carbolic acid, and about 2 c.c., or 30 minims, given to an ordinary adult animal. The United States and Canadian Governments each prepare it ready for use, but varying slightly in strength and dose. The size of the dose must be regulated for the condition, age, and sex of the animal. Adult males usually take a half more than females; very old animals more than medium; suspicious advanced cases should get a much larger dose; also re-tests within a short time should get a double dose.

Tuberculin is injected with an ordinary hypodermic syringe under the skin on the cervical, or just posterior to the scapular region, care being taken not to inject into the large lymphatic glands. Some directions give instructions to shave and disinfect the parts before injecting, but many of extensive experience say this is unnecessary. Unfavorable results so seldom follow that it will hardly compensate the trouble, unless it is in a herd which is unusually filthy or has some other disease present at the time.

The temperature must be taken once, and better two or three times before injecting, especially if the veterinarian is not thoroughly acquainted with the work. The time of injection makes no special difference, but much clearer reactions will be given if the test-period comes in the morning, when the animals are quiet and the diurnal variation is the lowest. From ten to twelve hours is the usual time given before the temperature is taken; then once in two hours until eighteen or twenty after the injection. If the injection is very early, one can wait as safely twelve hours as ten if a late injection is made, as the reaction seems to be a little quicker if the animal is up and feeding. Occasionally the reaction will not reach its height until nearly twenty-four hours after the injection, hence the necessity of varying the time according to the herd. A very close watch should be made of any suspicious animals during the test-period, as they usually show physical symptoms with the reaction. Some advanced cases give no temperature reaction, and then the veterinarian has to rely wholly upon physical reaction and symptoms. Animals may have a high temperature from other causes than the tuberculin; some of these are advanced stages of pregnancy, slight septicæmia following parturition, mammitis, laminitis, period of œstrum, indigestion from either over-feeding or feeding on straw and coarse foods, improper ventilation of the stable, sudden changes of atmospheric temperature. During the test the animals should be fed as usual, but no water should be allowed, as it may cause alterations of temperature at any time.

As there are so many irregularities which may complicate the testing, no hard-and-fast rule can be laid down for the amount of reaction to condemn an animal. The experience and sound judgment of the veterinarian is required to interpret the tuberculin-test in many instances. It is from the want of these two qualities on the part of the person making the test, and not the tuberculin, that is the cause of so many reported mistakes. The tuberculin-test will never be considered reliable, nor can it hold its present good standing as a diagnostic agent, if applied by the farmers themselves.

After condemning an animal with tuberculosis, the veterinarian must in most cases verify his diagnosis by a post-mortem examination. The owner of the animal usually removes the hide for its value, after which the veterinarian begins the autopsy. It is well to have a regular routine for such work, especially if there are hundreds of spectators. A very good method of procedure is something in this course:

1st. Support the carcass on its back.

2d. If a cow, examine the udder, supra-mammary glands, and remove.

3d. Cut sternum and linea alba, also abdominal muscles transversely.

4th. Examine peritoneum, omentum, small intestines, and mesenteric glands; remove abdominal portion of alimentary canal, and examine lymphatic glands on the stomach.

5th. Remove liver and hepatic glands, examine uterus and kidneys.

6th. Cut diaphragm and remove thoracic portion of trachea, lungs, and heart, and examine lungs, bronchial and mediastinal glands.

7th. Lastly, examine pre-scapular and retropharyngeal glands.

It would require too much time to describe the pathological appearances; furthermore, we are all more or less familiar with the descriptions. I will, however, speak of one form of the disease which may cause some one trouble: that is what is known as infiltration of tubercle. In these cases the characteristic nodule or tubercle is not found, but there is a diffuse inflammation through the organ affected, giving it an indurated feel. Upon microscopical examination numerous collections of cells may be seen, something similar to the beginning of a tubercle.

As bovine tuberculosis is of the most importance to the veterinarian, I will only briefly speak of it in other animals. Of the common domesticated animals the hog is, perhaps, the most subject to the disease after the ox. Quite often swine are affected where the cattle are badly diseased. Tuberculosis runs a much quicker course in hogs, and post-mortems show more of the softening and very little of the calcareous stage. Horses are occasionally affected; when seen it is usually more advanced in the intestines than lungs. Hens and dogs running in the barn with a badly infected herd sometimes contract the disease. Tuberculosis is very destructive to menageries of caged wild animals.

In closing, let us remember that the time is soon coming when there will be a more rigid examination of neat and beef-cattle in America; it therefore behooves us as young veterinarians to become thoroughly acquainted with tuberculosis, as well as other contagious diseases, in order to compete for appointments.

